# Development of the Common Cognitive Complaints after Concussion (C4) questionnaire: a treatment-planning tool for military service members and veterans with mild traumatic brain injury

**DOI:** 10.3389/fneur.2025.1621265

**Published:** 2025-07-23

**Authors:** Mollie Ann McDonald, Lisa H. Lu, Glenn Curtiss, Douglas B. Cooper, Amy O. Bowles, Melissa R. Ray, M. Marina LeBlanc, Blessen C. Eapen, Robert Shura, Lyn Turkstra

**Affiliations:** ^1^Mid-Atlantic Mental Illness Research, Education, and Clinical Center (MIRECC), Salisbury VAMC, Salisbury, NC, United States; ^2^Traumatic Brain Injury Center of Excellence (TBICoE), Military Health System, Silver Spring, MD, United States; ^3^Department of Rehabilitation, Brooke Army Medical Center, Houston, TX, United States; ^4^General Dynamics Information Technology, Falls Church, VA, United States; ^5^Independent Practice, Fort Myers, FL, United States; ^6^Polytrauma Rehabilitation Center, Audie L. Murphy VA Medical Center, San Antonio, TX, United States; ^7^Departments of Psychiatry and Rehabilitation Medicine, University of Texas Health Science Center at San Antonio, San Antonio, TX, United States; ^8^Division of Physical Medicine and Rehabilitation, David Geffen School of Medicine at UCLA, Los Angeles, CA, United States; ^9^Department of Physical Medicine and Rehabilitation, VA Greater Los Angeles Healthcare System, Los Angeles, CA, United States; ^10^Department of Neurology, School of Medicine, Wake Forest University, Winston-Salem, NC, United States; ^11^School of Rehabilitation Science, McMaster University, Hamilton, ON, Canada

**Keywords:** concussion, self-report, treatment planning, cognitive rehabilitation, cognition, occupational therapy, speech-language pathology

## Abstract

**Objective:**

To develop an activity-focused self-report tool to guide selection of treatment targets in cognitive rehabilitation for adults with mild traumatic brain injury (mTBI).

**Setting:**

Military and veteran treatment facilities.

**Participants:**

Twenty-one service members and 32 veterans with a history of mTBI; 25 veterans with orthopedic injury (OI).

**Design:**

Clinical tool development.

**Main measures:**

Common Cognitive Complaints after Concussion (C4) questionnaire.

**Results:**

We reviewed measures used in mTBI research or clinic, to identify items that could be used for selecting activity-level therapy targets as part of a treatment planning tool. To establish face and content validity, an initial item pool was reviewed by five speech-language pathology or occupational therapy mTBI experts who selected items relevant to their clinical practice, gave feedback on item wording, and suggested additional items. The result was a questionnaire with 22 activity-based items and one bias-check item. The C4 was then used in a feasibility mTBI treatment trial to identify treatment targets, and clinicians provided feedback on its utility. The C4 was also administered to an OI group to evaluate the distinctiveness of the items to mTBI symptoms.

**Conclusion:**

The C4 adequately captured activity-level functional impairments common to mTBI, and clinicians endorsed its utility as a useful tool to personalize treatment targets.

## Introduction

The lifetime prevalence of mild traumatic brain injury (mTBI), also known as concussion, is estimated to vary from 21 to 27% in the United States (1, 2). Most adults with mTBI recover without a need for treatment. However, an estimated 30% fail to recover spontaneously within 3 months (3) and seek rehabilitation for persistent cognitive complaints (4). Cognitive rehabilitation for adults with mTBI is symptom-focused, meaning treatment aims to reduce self-reported cognitive challenges in everyday life (5), regardless of whether symptoms are due to direct effects of neurotrauma. Consistent with evidence-based practice guidelines for rehabilitation after TBI (6), treatment is also individualized to the patient’s needs and priorities, and intended outcomes are performance improvements on everyday cognitively demanding tasks. The challenge for clinicians is identifying a measurement tool that captures these everyday cognitive tasks and can be used to help treatment planning.

There are several measurement tools designed to capture self-reported cognitive challenges after mTBI, these include the Neurobehavioral Symptom Inventory (NSI; (7)), Rivermead Post-Concussion Symptoms Questionnaire (8), Cognitive Failures Questionnaire (CFQ; (9)), and the PROMIS Item Bank for Cognitive Functions (10, 11). These questionnaires are widely used in outcome studies, but for the purpose of treatment planning they have several limitations. Scales such as the NSI and Rivermead were developed to capture all concussion symptoms, and thus include a mix of cognitive, somatic, and psychological symptoms with relatively few cognitive items. Scales like the CFQ have many cognitive questions but only in one domain (e.g., memory), and thus miss other cognitive symptoms. Many scales focus primarily at the impairment level of the International Classification of Functioning (12) rather than at the activity level (e.g., “Forgetfulness, poor memory” vs. “I have trouble keeping track when someone gives me a lot of details”) or have a mix of activity- and impairment-level items. There are existing questionnaires focused on activity limitations, such as the Behavior Rating Inventory of Executive Functions (13), but these were not developed for or standardized on adults with mTBI. Moreover, none of the aforementioned scales include follow-up questions necessary for treatment planning, such as questions about consequences of the problem in everyday life and how the person has attempted to manage it, and none ask examinees to choose what is most relevant for them and thus a priority for treatment. In sum, there is a need to develop a self-report questionnaire that captures cognitive challenges in their everyday contexts, includes cognitive activities typically affected by mTBI, and includes follow-up questions to provide guidance for intervention.

Here we describe the development of the Common Cognitive Complaints after Concussion (C4) questionnaire. The C4 is a self-report activity-based measure of everyday cognitive challenges. It was developed specifically for adults with mTBI and specifically for the purpose of functioning as a treatment planning tool as opposed to an outcome measure. The C4 was created and used within the context of Symptom-Targeted Approach to Rehabilitation for Concussion (STAR-C), a feasibility trial of cognitive rehabilitation for service members and veterans with cognitive complaints in the chronic stage after mTBI (25). As no single available questionnaire met the study’s needs, we set out to create a new instrument using an iterative process of expert consultation and consensus, then administered the instrument to service members and veterans with and without mTBI and conducted preliminary psychometric analyses.

## Materials and methods

### C4 development procedures

The first step in C4 development was to identify published scales that had been used in mTBI. We identified two validated scales from the research literature, the NSI (7) and Everyday Memory Questionnaire (14); a third that was published in a textbook (26) and recommended by colleagues; and a fourth that was a component of a published treatment protocol (15). Each had items relevant for mTBI treatment planning, but no single scale focused on cognitive activities and was validated in mTBI, and there was considerable overlap among scales. Thus, the group began by identifying scale items that were relevant to clinical practice and eliminating duplicates, then mapped scale items to cognitive domains to identify areas of overlap and gaps. This version was sent to five mTBI speech-language pathology (SLP) or occupational therapy (OT) experts in the Department of Defense (DoD), Veterans Administration (VA), and academic settings across the U.S.

SLP and OT experts provided item-level feedback and recommendations for additional items and/or wording changes (e.g., phrasing all items as “I” statements). Feedback was aggregated and a revised version was sent back to experts for review in two subsequent rounds. After the final round, all five experts agreed for the inclusion of the selected items. In addition to item-level feedback, experts provided suggestions regarding questionnaire instructions and follow-up interview questions to facilitate using the questionnaire specifically as a treatment planning tool. We incorporated this feedback, for example, specifying in the instructions that identified challenges should be “*new* since [examinees’] injury and affect [their] everyday functioning” because experts emphasized the clinical utility of identifying changes from baseline. In addition, we included interview questions to be asked post-questionnaire, which experts identified would facilitate the treatment planning process. These included clarifying questions for items rated as frequently bothersome (e.g., “Can you give me an example of this happening in your everyday life?”), as well as a question asking examinees to identify their top three greatest challenges, as a means of identifying treatment targets and addressing possible negative bias (i.e., identifying their greatest challenges even if participants identify several items as frequently bothersome). The initial list of 49 items with sources is shown in the Supplementary Appendix with initial expert feedback (i.e., initial qualitative ratings of “possibly include” and “possibly exclude”). The final 23-item version with instructions and follow-up interview questions is shown in Appendix A. The final version of the C4 included 22 items evaluated everyday complaints commonly reported in the chronic mTBI population, and one item was a bias check (item 16). The bias-check item was selected to be a non-declarative memory task that would be unaffected by mTBI.

A separate group of five mTBI experts, including physicians and neuropsychologists, sorted the items into cognitive domains to assess content validity. These experts were not given domain categories in advance; instead, they each generated their own domain categories. Items were assessed for consensus across experts to determine if cognitive domains were adequately covered.

### Participants

The C4 was administered to service members with a history of mTBI (*n* = 21), veterans with a history of mTBI (*n* = 32), and veterans with orthopedic injuries but no history of mTBI (*n* = 25). Table 1 presents demographic and clinical characteristics for the full sample (*N* = 78). Participants were recruited from a military treatment facility and a VA Polytrauma/TBI System of Care program.

**Table 1:** Participant demographic and clinical characteristics.

Variable	MTBI		Orthopedic injury *n* = 25
	Active duty *n* = 21	Veteran *n* = 32	
Age in years, ^*^ mean (*SD*)	37.57 (8.06)	47.56 (9.42)	53.32 (10.54)
Female, *n* (%)	5 (23.8)	10 (31.3)	6 (24.0)
Race, *n* (%)			
White	14 (66.7)	14 (43.8)	10 (40)
Other	7 (33.3)	18 (56.3)	15 (60)
Hispanic, *n* (%)	4 (19.0)	13 (40.6)	9 (36.0)
Marital status, *n* (%)			
Married	16 (76.2)	23 (71.9)	15 (60.0)
Not married	5 (23.8)	9 (28.1)	10 (40.0)
Education^a^, *n* (%)			
< High school	1 (5.0)	0 (0.0)	0 (0.0)
High school	2 (10.0)	3 (9.4)	6 (24.0)
> High school	17 (85.0)	29 (90.6)	19 (76.0)
Years of military service^a,*^ mean (*SD*)	16.95 (8.33)	16.00 (9.31)	10.50 (8.26)
OEF/OIF^b^ deployments^a,*^, *n* (%)			
0	6 (30.0)	5 (15.6)	12 (48.0)
1	4 (20.0)	10 (31.3)	9 (36.0)
2+	10 (50.0)	17 (25.0)	4 (8.0)
Clinical characteristics			
Lifetime TBIs reported, *n* (%)			N/A
1	8 (38.1)	10 (31.3)	
2+	13 (61.9)	22 (68.7)	
Months since most recent TBI^**^, mean (*SD*)	87.53 (69.65)	150.72 (81.89)	N/A
AOC^c^, *n* (%)			N/A
None	4 (19.0)	4 (12.5)	
Positive AOC	17 (23.8)	28 (28.1)	
Chronic pain, *n* (%)	16 (76.2)	14 (43.8)	16 (64.0)
Prior mental health condition^*^, *n* (%)	9 (42.9)	6 (18.8)	13 (52.0)
Current PTSD^***^, *n* (%)	9 (42.9)	24 (75.0)	2 (8.0)
Current other anxiety disorder^***^, *n* (%)	14 (66.7)	19 (59.4)	3 (12.0)
Current mood disorder^***^, *n* (%)	11 (52.4)	20 (62.5)	3 (12.0)
Current ADHD, *n* (%)	2 (9.5)	3 (9.4)	1 (4.0)
Current alcohol/substance abuse/dependence, *n* (%)	2 (9.5)	6 (18.8)	0 (0.0)

Statistical tests included use of contingency tables, *t*-tests, or ANOVAs depending on the level of measurement of the variable. All participants lived in the southwest region of the U.S. ^a^Excludes those who did not respond to the question. ^b^OEF/OIF = Operation Enduring Freedom/Operation Iraqi Freedom. ^c^AOC = Alteration of consciousness. ^*^p < 0.05. ^**^p < 0.01. ^***^p < 0.001.

Participants with mTBI were recruited as part of STAR-C. Mild TBI was determined based on clinical judgment of a medical provider embedded within a clinic specialized for TBI care and defined using the VA/DoD criteria (16): 30 min or less of loss of consciousness, less than 24 h of alteration of consciousness, or 1 day or less of posttraumatic amnesia; and no abnormalities on neuroimaging, if available. The mTBI must have occurred at least 6 months prior to the participant starting the study. Clinical determination was based on both self-report and medical record review. Participants must have endorsed at least one cognitive complaint as “moderate” or more severe on the NSI (7) and demonstrated at least a 6th grade reading level on the Wide Range Achievement Test, 4th Edition, Word Reading subtest (WRAT-4 Reading; (17)) to qualify for STAR-C. Exclusion criteria included a history of moderate, severe, or penetrating TBI; a lifetime diagnosis of schizophrenia or other psychotic disorder; a history of neurological disease (e.g., multiple sclerosis, cerebrovascular accident); active suicidal ideation; current participation in intensive behavioral health treatment, defined as more than 5 appointments/encounters per week; inability to attend treatment three times per week for 3 weeks; use of daily narcotic medication; or failure on measures of performance validity, per the Test of Memory Malingering (18) manual cutoff score or symptom validity, per the NSI Validity-10 cutoff score (19).

Participants in the orthopedic injury group were recruited from the Polytrauma Musculoskeletal and Orthopedic Injury clinic at the same VA hospital. Reasons for orthopedic treatment included traumatic orthopedic injuries, wear-and-tear injuries, and chronic pain. Individuals who indicated a history of mTBI during prescreening were not invited into the study. Medical records of enrolled participants were reviewed to confirm a lack of documented mTBI. Time since injury was calculated from the date of the presenting orthopedic/musculoskeletal injury to date of enrollment.

Table 1 presents demographic and clinical characteristics of our sample, separated by groups of service members with a history of mTBI, veterans with mTBI, and veterans with orthopedic injuries. We statistically compared the groups using contingency tables, *t*-tests, or ANOVAs depending on the variables’ level of measurement. These tests were conducted to ensure the samples were representative of what is typical for their respective population (e.g., to ensure the orthopedic group presented with fewer mental health conditions than the mTBI groups). The three samples differed in age, with service members being the youngest and the orthopedic sample the oldest. Participants with orthopedic injuries had fewer years of military service and fewer Operation Enduring Freedom/Operation Iraqi Freedom combat deployments than those with mTBI. Fewer veterans with a history of mTBI also had a history of prior mental health conditions than the other two samples. Veterans with a history of mTBI had the highest current posttraumatic stress disorder (PTSD) rate overall, which was higher than the current PTSD rate in the orthopedic sample. Participants with orthopedic injuries also had fewer other anxiety disorders than the two samples with a history of mTBI, and the fewest mood disorder diagnoses, with the most being in the veteran sample with a history of mTBI.

### Measures

The C4 questionnaire was presented to participants in the format shown in Appendix A, which includes instructions to participants. A 5-point frequency of occurrence scale (“Not at all” to “All the time”) was used to rate an item’s persistence over a 2-week period. For items rated as “Often” or “All the time,” clinicians asked the additional questions, “In what setting is this most disruptive (e.g., home, school, community, work)? Can you give me an example of this happening in your everyday life? What do you do when it happens? What physical or psychological factors influence this happening and how you deal with it?” These follow-up questions were intended to assist clinicians and participants in identifying the contexts in which difficulties tend to occur, concrete examples of cognitive difficulties in daily life, existing coping strategies, as well as perceived causes of these difficulties. Participants were then asked to identify the top three items from the questionnaire that represented the most bothersome functional limitations. For the veteran and active-duty mTBI groups, these three items were the treatment targets which served as the starting point for STAR-C therapy.

In addition to the C4, participants completed the Minnesota Multiphasic Personality Inventory – 3 (20). The MMPI-3 is a 335-item, true-false, self-report measure of psychopathology. It yields ten validity scales/indicators, three higher-order scales, eight substantive restructured clinical scales, 26 specific problems scales, and five personality psychopathology scales. The specific problems scales of Cognitive Complaints and Inefficacy (e.g., decision-making difficulties) were chosen to evaluate the C4’s convergent validity, given that these scales also capture subjective cognitive complaints.

### Data collection procedures

After providing written informed consent to participate, all participants completed a demographic data questionnaire, the C4, and other questionnaires relevant to the larger STAR-C feasibility trial. Participants with a history of mTBI also completed other procedures as part of STAR-C (further details are provided in Turkstra et al. (25); only relevant details are summarized here). If a participant was not able to identify at least three C4 items that occurred “Often” or “All the time,” they were to be excluded from the study; however, all eligible participants could identify three problem items on the C4 at intake, so none were excluded. Participants who advanced to treatment collaborated with clinicians to reframe C4 problem statements as treatment targets (e.g., “I get overwhelmed by things I have to do” might be reframed as “Each morning, I will choose three tasks to complete that day”). Participants worked to attain those targets in 6–10 individual strategy-focused cognitive rehabilitation sessions within 4 weeks. Treatment was discontinued when the participant had attained three treatment targets or completed 10 sessions, whichever came first. Target attainment was measured using goal attainment scaling (21), and was measured immediately after therapy and at 1 and 3 months post-therapy. In addition to patient data, we informally solicited feedback from the clinicians who administered the C4 regarding the C4’s ease of use and clinical utility. Moreover, the C4 was re-administered to participants with orthopedic injuries approximately 4 weeks after the first administration, and we used these data to evaluate test–retest stability of the instrument because scores were not expected to change over time for this group given that they received no treatment. No participants within the orthopedic injury group were lost to follow-up. All procedures involving human subjects were approved by the Institutional Review Board for human participants’ protection at both enrolling sites.

### Data analysis plan

Given that the C4 was developed as a treatment planning tool and not an assessment tool, our planned analyses focused on demonstrating the clinical use of the C4 and not on demonstrating a breadth of psychometric properties. For treatment planning, the essential psychometric properties are face validity (e.g., items capture functions that patients and clinicians judge as relevant to therapy) and content validity (e.g., items are based on a conceptual framework and capture appropriate content domains). The face and content validity of the C4 are largely reflected in our scale development procedures, such that the items for the C4 were developed and vetted by outside clinicians not involved in the STAR-C study. We also calculated percentages of item endorsement frequences among our three groups (service members with TBI, veterans with TBI, veterans with orthopedic injury) to determine if C4 items covered content that was relevant and specific to mTBI functional outcomes as opposed to generalized functional outcomes that would be expected in any group with a history of injury (e.g., orthopedic injury). We reported relative differences in endorsements as opposed to statistical differences due to Type I error inflation and power concerns within the context of the STAR-C feasibility trial’s small sample. To demonstrate the clinical utility of the C4, we included information regarding STAR-C outcomes; specifically, we reported the percentages of participants who successfully attained treatment goals at post-treatment and follow-up data collection time points. Additionally, though a formal qualitative analysis was not conducted, we included excerpts of the qualitative information provided by STAR-C clinicians regarding their perceptions of the C4 as a treatment planning tool.

Importantly, though it was not the focus of the current study to demonstrate the psychometric properties of the C4 as if it were an assessment tool, we conducted basic psychometric analyses to provide preliminary evidence of its psychometric soundness. Specifically, the factor structure of the C4 was evaluated using exploratory factor analysis (EFA) in the mTBI samples only (*n* = 53). The bias-check item was excluded from EFA procedures because this item was intentionally designed to represent a cognitive task that would be unaffected by mTBI (i.e., a non-declarative memory task) and therefore would not be expected to load with other items. Item scores were submitted to principal-axis factor analysis with squared multiple correlations as the initial communality estimates. Items were judged to load on a factor if the highest factor loading was ≥0.400 (22), and cross-loadings were resolved by assigning an item to the factor with the stronger loading. In addition, we assessed reliability using internal consistency and stability measures. Internal consistency was evaluated within factors by calculating Cronbach’s alpha. Stability was evaluated by calculating test–retest reliability correlation coefficients on the data obtained from the orthopedic injury sample (*n* = 25) at two timepoints: baseline and 4 weeks later. Validity was examined by using data from the mTBI sample (*n* = 53) by correlating factor scores with selected MMPI-3 problems scales. It was predicted that convergent validity would be demonstrated by significant positive correlations with Cognitive Complaints and Inefficacy.

## Results

### Domain coverage

Content domains are reported in Table 2. Out of the five experts tasked with sorting the 23 C4 items into cognitive domains, at least 80% agreed that 4 items captured the attention domain, 3 items captured the memory domain, and 3 items captured the processing speed domain. At the level of 60% or more agreement, 4 items were agreed to capture the attention domain, 7 items captured the memory domain, 5 items captured the processing speed domain, and 1 item captured the executive functioning domain. Therefore, in total, five experts agreed on the content sorting for 17 out of 23 items at the level of 60% or more agreement, and they agreed upon the content categories for 10 items at the level of 80% or more agreement. Notably, these four content categories were generated by the experts themselves.

**Table 2:** Content domains as determined by expert consensus.

Cognitive domain	Item numbers	
	≥80% Agreement	≥60% Agreement
Attention	3,4,13,15	3,4,13,15
Memory	1,7,12	1,7,12,16,20,22,23
Processing speed	5,14,21	2,5,10,14,21
Executive functioning		8

Five experts sorted the 23 C4 items into self-generated cognitive domains.

### C4 item endorsement frequencies

**Figure 1:**
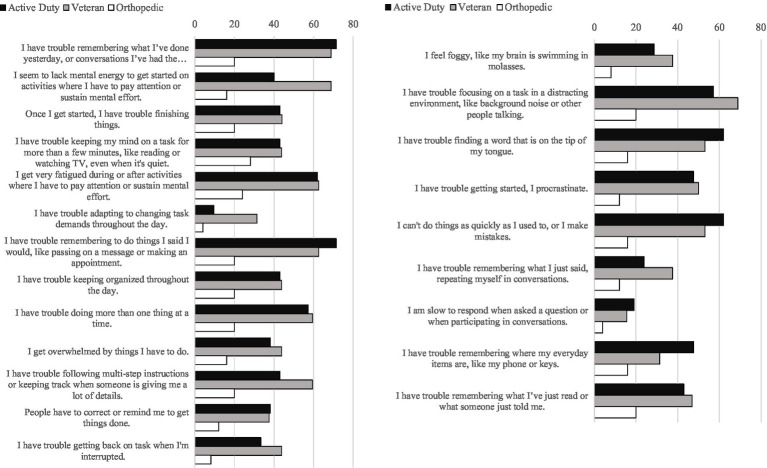
Percent of C4 items endorsed “often” or “all the time” by each group.

See Appendix A for a list of all items as they were presented to participants. Specifically, Figure 1 depicts the percentage of each item endorsed as “Often” or “All the time” by each group, while all item endorsement frequencies are presented in more detail in Supplementary Table 1. All items were endorsed as being problematic “Often” or “All the time” relatively more frequently by the mTBI samples than by those with orthopedic injuries. Seven problems were endorsed by more than 50% of both mTBI samples as occurring “Often” or “All the time.” In order from most to least frequent, these were: (1) “I have trouble remembering to do things I said I would, like passing on a message or making an appointment.” (2) “I have trouble remembering what I’ve done yesterday, or conversations I’ve had the previous day.” (3) “I cannot do things as quickly as I used to, or I make mistakes.” (4) “I have trouble finding a word that is on the tip of my tongue.” (5) “I get very fatigued during or after activities where I have to pay attention or sustain mental effort.” (6) “I have trouble focusing on a task in a distracting environment, like background noise or other people talking.” (7) “I have trouble doing more than one thing at a time.” Notably, our bias check (item 16) was endorsed as being problematic “Often” or “All the time” by only one participant out of our entire sample of 78 participants, reflecting its utility as an infrequently endorsed item.

### STAR-C C4 treatment target identification results

STAR-C outcomes are described in detail in Turkstra et al. (25). In brief, of the 53 mTBI participants who completed STAR-C, 51 (96.23%) successfully attained at least one of their three identified targets at the end of treatment, with 81% successfully attaining all three. Of the 51 who successfully attained targets, ratings were available for 40 participants at 4 weeks and 12 weeks post-treatment. Of those 40, 80% continued to successfully attain at least one of the three C4-identified target areas at both follow-ups.

### Clinician feedback

STAR-C clinicians reported that the C4 helped participants talk about their cognitive complaints and how those complaints translated into specific functional challenges. Clinicians noted that it was helpful to have specific daily living task examples for participants to reflect on as they described the impact of their cognitive challenges in everyday life. As one clinician noted, patients often reported concerns with ‘memory’ and ‘attention’ but were unable to expand on those concerns. The C4 questions provided practical terminology to help participants express how their cognitive difficulties affected everyday functioning, which then transferred to identifying meaningful targets. As one clinician said, the C4 allowed a “seamless transition” from assessment to treatment. Clinicians noted that patients seemed to “resonate with” the questions, and said that the questions put their thoughts into words (e.g., a participant reportedly said that “it’s like you were reading my mind”), which was an “immediate in” for the therapeutic relationship.

### Preliminary psychometric properties

According to EFA, eigenvalues for the first three factors in the unrotated solution were: 7.187, 2.246, and 1.692; and accounted for 30.34, 7.62, and 5.29 percent of the variance, respectively, for a total variance accounted for of approximately 43 percent. The final varimax-rotated solution is presented in Table 3, and descriptive statistics for each factor are presented in Table 4. We chose to label factors using terms that describe functional impact to align with our goal for the C4 to identify activity-based treatment targets. We labeled our factors *Keeping Attention Focused* (9 items; e.g., “I have trouble doing more than one thing at a time”), *Seeing Things Through* (7 items; e.g., “Once I get started, I have trouble finishing things”), and *Being Present* (4 items; e.g., “I have trouble remembering what I just said, repeating myself in conversations”). To confirm the appropriateness of the three-factor solution, we correlated unit-weighted scores for the three factors (see Table 4). Correlations ranged from 0.327 to 0.656 (*p*s < 0.05), suggesting that these three factors are related yet separate constructs (i.e., positively correlated but not very strongly correlated), thereby providing additional support for the three-factor solution.

**Table 3:** C4 varimax-rotated factor structure.

C4 Item		Factor loading		
		1. *Keeping Attention Focused*	2. *Seeing Things Through*	3. *Being Present*
1	I have trouble remembering what I’ve done yesterday, or conversations I’ve had the previous day.	0.258	**0.471**	0.377
2	I seem to lack mental energy to get started on activities where I have to pay attention or sustain mental effort.	0.333	**0.632**	0.090
3	Once I get started, I have trouble finishing things.	0.434	**0.743**	−0.030
4	I have trouble keeping my mind on a task for more than a few minutes, like reading or watching TV, even when it’s quiet.	**0.534**	0.522	−0.082
5	I get very fatigued during or after activities where I have to pay attention or sustain mental effort.	**0.426**	0.416	0.151
6	I have trouble adapting to changing task demands throughout the day.	**0.623**	0.256	0.013
7	I have trouble remembering to do things I said I would, like passing on a message or making an appointment.	0.054	**0.632**	0.352
8	I have trouble keeping organized throughout the day.	0.396	**0.565**	0.013
9	I have trouble doing more than one thing at a time.	**0.747**	0.093	0.128
10	I get overwhelmed by things I have to do.	**0.551**	0.285	0.087
11	I have trouble following multi-step instructions or keeping track when someone is giving me a lot of details.	**0.603**	0.076	0.500
12	People have to correct or remind me to get things done.	0.276	**0.497**	0.482
13	I have trouble getting back on task when I’m interrupted.	**0.644**	0.203	0.251
14	I feel foggy, like my brain is swimming in molasses.	**0.545**	0.243	0.117
15	I have trouble focusing on a task in a distracting environment, like background noise or other people talking.	**0.514**	0.097	0.316
17	I have trouble finding a word that is on the tip of my tongue.	0.159	0.080	0.327
18	I have trouble getting started, I procrastinate.	0.095	**0.601**	0.052
19	I cannot do things as quickly as I used to, or I make mistakes.	0.138	0.263	**0.493**
20	I have trouble remembering what I just said, repeating myself in conversations.	0.058	−0.036	**0.686**
21	I am slow to respond when asked a question or when participating in conversations.	−0.016	0.017	**0.476**
22	I have trouble remembering where my everyday items are, like my phone or keys.	0.050	0.340	0.049
23	I have trouble remembering what I’ve just read or what someone just told me.	0.127	0.071	**0.576**

Items were assigned to factors based on the highest factor loading ≥ 0.400. Bolded factor loadings indicate factor scale assignment.

**Table 4:** Descriptive statistics and correlations.

Variable	*M*	*SD*	*Seeing Things Through*	*Being Present*	Cognitive complaints	Inefficiency
*Keeping Attention Focused*	30.472	6.758	0.656 (<0.001)	0.327 (0.017)	0.421 (0.002)	0.592 (<0.001)
*Seeing Things Through*	24.887	5.048	–	0.333 (0.015)	0.434 (0.001)	0.419 (0.002)
*Being Present*	12.906	2.726	–	–	0.175 (0.210)	0.184 (0.187)
Cognitive complaints	70.717	9.945	–	–	–	–
Inefficiency	53.623	10.244	–	–	–	–

*N* = 53. *M* = mean; *SD* = standard deviation. Cognitive complaints and inefficiency, are MMPI-3 scales and thusly are interpretable as T-scores (*M* = 50; *SD* = 10) with scores of *T* ≥ 65 generally being considered clinically elevated. *Keeping Attention Focused*, *Seeing Thing Through*, *and Being Present* are C4 factor scores. Correlations are Pearson product–moment r correlation coefficients (*p*-values).

The *Keeping Attention Focused* and *Seeing Things Through* factors showed homogenous construct measurement, *r*_α_s = 0.859 and 0.851, respectively. The *Being Present* factor had an internal reliability coefficient of 0.636, reflecting the small number of items comprising this scale. Moreover, though alpha values >0.70 are preferred, values >0.60 may be considered acceptable for the construction of new measures (23). All three factor scales showed high stability across a 4-week period among the orthopedic group (*p*s < 0.001): *Keeping Attention Focused r* = 0.785, *Seeing Things Through r* = 0.783, and *Being Present r* = 0.871.

As presented in Table 4, the C4 factors overall showed positive correlations with Cognitive Complaints and Inefficacy scales, consistent with hypotheses related to convergent validity. Specifically *Keeping Attention Focused* and *Seeing Things Through* were moderately positively correlated with Cognitive Complaints and Inefficacy (*r*s = 0.419–0.592, *p*s ≤ 0.002). Results were not as consistent with our hypotheses for *Being Present*, such that relationships with Cognitive Complaints and Inefficacy were positive, yet weaker and nonsignificant (*r*s = 0.175–0.184, *p*s ≥ 0.187). Notably, the third factor retained, *Being Present*, was the weakest in terms of eigenvalue, number of items, internal reliability, and convergent validity. Yet, several observations reinforced our decision to retain it. This factor was weakly positively correlated with Cognitive Complaints and Inefficiency, a pattern providing some support for the validity of this factor as an area of cognitive complaint in individuals with a history of mTBI. Moreover, despite having only four items that loaded onto this factor, one of its items was among the most frequently endorsed within our sample, “I cannot do things as quickly as I used to, or I make mistakes.” This item was endorsed as a problem “Often” or “All the time” by over half of both the military and veteran mTBI samples (see Figure 1). Therefore, this level of endorsement supported *Being Present* as an area of concern that warrants clinical attention in cognitive rehabilitation for individuals with a history of mTBI. Thusly, we retained *Being Present* as a factor.

Relatedly, two items, “I have trouble finding a word that is on the tip of my tongue” and “I have trouble remembering where my everyday items are, like my phone or keys,” did not load on any factors. We retained these items in the C4 given the C4’s original purpose as a treatment planning tool. These items are still useful for treatment planning purposes, such that 61.9 and 47.6% of active duty and 53.1 and 31.3% of veteran military service members with a history of mTBI endorsed, respectively, difficulties with word-finding and misplacing items at the levels of “Often” or “All the time” (See Figure 1).

## Discussion

Though a variety of self-report questionnaires are available for evaluating outcomes relevant to mTBI, clinicians need tools specifically to support intervention planning, particularly when current practice guidelines (6) emphasize the importance of focusing on improving performance in activities rather than attempting to remediate impairments. The C4 was developed to meet that need, to help clinicians and patients identify treatment targets in cognitive rehabilitation after mTBI. Consensus from experts as well as findings from a sample of service members and veteran participants with a history of mTBI supported the face and content validity of the C4 as a survey of cognitive complaints relevant to mTBI. In addition, outcome results and clinician feedback from STAR-C, a feasibility trial of cognitive rehabilitation for service members, support its clinical utility as a treatment planning tool, and psychometric analyses provide preliminary support for the psychometric soundness of the C4.

Given that the C4 was developed specifically as a treatment tool, we focused on establishing its face and content validity. We initially addressed validity through an iterative process of item development with expert input from professionals who administer cognitive rehabilitation interventions (i.e., speech language pathologists and occupational therapists). Then, a separate group of mTBI experts, including physicians and neuropsychologists, sorted items by cognitive domain. Importantly, this confirmed an intended purpose of the C4: to capture multiple cognitive domains relevant to mTBI. Specifically, expert consensus confirmed the C4’s coverage of four categories (i.e., attention, memory, processing speed, and executive functioning) which represent the four cognitive domains most functionally impacted by mTBI (24).

Beyond feedback from experts, we also assessed the C4’s item content using EFA. Though factors derived from EFA did not align with the areas of content identified by experts, the presence of three EFA-derived factors provides further support for the C4 as a measure of multiple cognitive domains relevant to mTBI. Moreover, given that the C4 measured cognitive difficulties in terms of activity-based items as opposed to domain-based items, the factors were labeled at the activity level: *Keeping Attention Focused*, *Seeing Things Through*, and *Being Present*. Internal consistency within factors and stability across a 4-week period were shown to be good, and correlations with related constructs (i.e., other measures of subjective cognitive complaints) were consistent with convergent validity.

In addition to expert consensus and factor analysis, the C4’s content validity was also supported by participant responses. Endorsement of C4 items across many participants demonstrated the universality of complaints reported by participants with a history of mTBI. Moreover, the high frequency of item endorsement in the mTBI samples indicated that items represented problems of sufficient frequency that they could be used to identify treatment targets. All potential participants also could identify at least three items on the C4 that were problems for them in daily living, thereby supporting the C4’s clinical utility. Although activity-based cognitive problems were endorsed by all groups in the study, including the orthopedic injury group, C4 items were more frequently endorsed by individuals who reported a history of mTBI. Taken together, these data provide preliminary support for C4’s coverage of common cognitive problems that bring individuals with mTBI into treatment.

In further support of the C4’s clinical utility, findings from the STAR-C treatment trial suggested that the C4 successfully helped participants and therapists identify individualized targets for treatment. Participants successfully achieved therapy targets and a significant proportion continued to successfully meet those targets for at least 3 months post-treatment. Given the brevity of treatment (a maximum of 10 individual sessions over 3–4 weeks), there was a need for a treatment planning tool to efficiently help participants identify activity-based treatment targets. Qualitative feedback from STAR-C clinicians supported the utility of the C4 in this manner. According to feedback, the C4 allowed for a seamless transition to developing strategies and addressing everyday limitations associated with cognitive complaints.

### Limitations and future directions

Despite support regarding the C4’s utility as a treatment planning tool, the current study is not without limitations. Several of the current study’s limitations were due to data being collected as part of a mTBI treatment demonstration project (STAR-C; 25) where the C4 was the clinical instrument used for identifying intervention targets among military service members beginning cognitive rehabilitation. Ultimately, the C4 was not originally intended to be assessed as a potential outcome measure. Therefore, one major limitation of this study was the small sample sizes as well as our restriction to military service member samples, whose responses might not generalize to the general population. Future research may wish to use a larger and more diverse sample to test if sociodemographic factors influence C4 responses. A larger sample would also allow for examining more complex relationships between variables, such as with the use of methods like structural equation modeling.

Moreover, though we present preliminary psychometric analyses, including a tentative factor structure for this instrument, the results should be replicated with a much larger sample and using additional analytic methods, such as confirmatory factor analysis. Furthermore, though we present preliminary qualitative feedback from clinicians, the current results could be replicated with a larger sample of clinicians and/or with a structured interview to elicit more qualitative data from clinicians to facilitate the use of formal content analyses to increase the objectivity of qualitative data analysis. Future research may also wish to include an additional uninjured control group to test if the difficulties captured by the C4 reflect general difficulties unrelated to injury. Lastly, for the purposes of STAR-C, potential participants who demonstrated performance validity or symptom validity failure were intentionally excluded and therefore did not complete the C4. Future research could compare responses to the C4’s bias-check item with established symptom validity measures to establish a cutoff score that corresponds with symptom validity pass versus failure. This, as well as other future research addressing the aforementioned limitations, would bolster the C4’s utility as a brief, activity-focused outcome measure tailored to capturing common cognitive complaints among adults with mTBI.

As a means of bolstering the C4’s utility as a treatment planning tool, future research may consider adaptations. For example, two separate ratings, one for pre-injury and one for post-injury, could help patients to reflect on their baseline cognitive abilities in a manner that is not idealized, and thereby suggest that the end goal of cognitive rehabilitation is not to function perfectly. Moreover, C4 items could be further refined using strategies such as the inclusion of additional response options (e.g., “Not sure” or “Not applicable”) which would allow for the identification and subsequent rephrasing or removal of confusing or unclear items. Additional items could be developed with the inclusion of a patient-generated item (e.g., “Is there something you are struggling with that is not included in this survey?”) that would allow for the identification of additional often-reported functional difficulties. Lastly, while evidence suggests that the C4 is a useful clinical tool for the purposes of treatment plan development to learn compensatory strategies through cognitive rehabilitation, it is also important to note that a holistic approach to mTBI treatment might be necessary to address comorbid conditions (e.g., chronic pain, sleep disturbances, mental health difficulties) that can contribute to cognitive inefficiencies in daily life as opposed to solely focusing on compensatory strategies. Notably, participant’s responses to the C4’s follow-up question, “What physical or psychological factors influence this [cognitive difficulty] happening and how you deal with it?” could provide insight into what additional interventions might be needed to address a patient’s symptoms holistically (e.g., psychotherapy if cognitive difficulties tend to increase when under emotional stress). A holistic approach may be especially indicated if some patients are found to not respond as well to cognitive rehabilitation protocols, like STAR-C, as compared to other patients.

## Conclusion

There are many self-report tools for evaluating patients with mTBI, including tools sampling a variety of domains and tools focusing on cognitive impairments. These tools have been used successfully to describe clinical samples and measure outcomes after injury, but they were not designed for treatment planning. The C4 was developed specifically to help clinicians efficiently identify activity-based treatment targets in collaboration with patients. It was designed as a guide for beginning treatment and a foundation for clinical reasoning about treatment targets. Results of the present study and STAR-C suggest that the C4 functions well in this role. Though further evaluation and validation is warranted, evidence supports the clinical use of C4 as a survey tool as well as preliminary support of its psychometric properties.

## Data Availability

The raw data supporting the conclusions of this article will be made available by the authors, without undue reservation.
