# Supplementary material for: Development of the Common Cognitive Complaints after Concussion (C4) questionnaire: a treatment-planning tool for military service members and veterans with mild traumatic brain injury
Source: Front Neurol. 2025 Jul 23;16:1621265. doi: 10.3389/fneur.2025.1621265 (PMC12325049; doi:10.3389/fneur.2025.1621265)
Supplement: Supplementary file 1 [file Data_Sheet_1.docx]

## Appendix A

*Common Cognitive Complaints after Concussion (C4) questionnaire*

Instructions: We are going to ask you about some everyday thinking challenges. All of us experience the sorts of challenges on this list, mTBI or not. Our goal is to identify challenges that are *new* since your injury and affect your everyday functioning, so we can focus therapy on what is most important to you. For each question, choose the number that shows how often this has caused problems for you in everyday life in the past two weeks.

|  | Question | Not at all | Rarely | Sometimes | Often | All the time |
| --- | --- | --- | --- | --- | --- | --- |
| 1 | I have trouble remembering what I did yesterday, or conversations I’ve had the previous day. | 1 | 2 | 3 | 4 | 5 |
| 2 | I seem to lack mental energy to get started on activities where I have to pay attention or sustain mental effort. | 1 | 2 | 3 | 4 | 5 |
| 3 | Once I get started, I have trouble finishing things. | 1 | 2 | 3 | 4 | 5 |
| 4 | I have trouble keeping my mind on a task for more than a few minutes, like reading or watching TV, even when it's quiet. | 1 | 2 | 3 | 4 | 5 |
| 5 | I get very fatigued during or after activities where I have to pay attention or sustain mental effort. | 1 | 2 | 3 | 4 | 5 |
| 6 | I have trouble adapting to changing task demands throughout the day. | 1 | 2 | 3 | 4 | 5 |
| 7 | I have trouble remembering to do things I said I would, like passing on a message or making an appointment. | 1 | 2 | 3 | 4 | 5 |
| 8 | I have trouble keeping organized throughout the day. | 1 | 2 | 3 | 4 | 5 |
| 9 | I have trouble doing more than one thing at a time. | 1 | 2 | 3 | 4 | 5 |
| 10 | I get overwhelmed by things I have to do. | 1 | 2 | 3 | 4 | 5 |
| 11 | I have trouble following multi-step instructions or keeping track when someone is giving me a lot of details. | 1 | 2 | 3 | 4 | 5 |
| 12 | People have to correct or remind me to get things done. | 1 | 2 | 3 | 4 | 5 |
| 13 | I have trouble getting back on task when I'm interrupted. | 1 | 2 | 3 | 4 | 5 |
| 14 | I feel foggy, like my brain is swimming in molasses. | 1 | 2 | 3 | 4 | 5 |
| 15 | I have trouble focusing on a task in a distracting environment, like background noise or other people talking. | 1 | 2 | 3 | 4 | 5 |
| 16 | I forget how to do routine activities like driving a car. | 1 | 2 | 3 | 4 | 5 |
| 17 | I have trouble finding a word that is on the tip of my tongue. | 1 | 2 | 3 | 4 | 5 |
| 18 | I have trouble getting started, I procrastinate. | 1 | 2 | 3 | 4 | 5 |
| 19 | I can't do things as quickly as I used to, or I make mistakes. | 1 | 2 | 3 | 4 | 5 |
| 20 | I have trouble remembering what I just said, repeating myself in conversations. | 1 | 2 | 3 | 4 | 5 |
| 21 | I am slow to respond when asked a question or when participating in conversations. | 1 | 2 | 3 | 4 | 5 |
| 22 | I have trouble remembering where my everyday items are, like my phone or keys. | 1 | 2 | 3 | 4 | 5 |
| 23 | I have trouble remembering what I’ve just read or what someone just told me. | 1 | 2 | 3 | 4 | 5 |

*Note*. For treatment planning, self-administration of the questionnaire is followed by an interview in which, for items rated as Often/All the time, the clinician asks the following questions: In what setting is this most disruptive (e.g., home, school, community, work)? Can you give me an example of this happening in your everyday life? What do you do when it happens? What physical or psychological factors influence this happening and how you deal with it? In addition, after examinees have completed the questionnaire, they are asked to identify the top 3 challenges in their everyday lives from among the scale items as a means of identifying treatment targets.
