# Supplementary material for: Development of the Common Cognitive Complaints after Concussion (C4) questionnaire: a treatment-planning tool for military service members and veterans with mild traumatic brain injury
Source: Front Neurol. 2025 Jul 23;16:1621265. doi: 10.3389/fneur.2025.1621265 (PMC12325049; doi:10.3389/fneur.2025.1621265)
Supplement: Supplementary file 2 [file Table_1.docx]

## Supplementary Table 1

*C4 item endorsement frequencies by clinical group*

| C4 item | Active Duty (*n* = 21) | | | Veteran (*n* = 32) | | | Orthopedic Injury (*n* = 25) | | |
| --- | --- | --- | --- | --- | --- | --- | --- | --- | --- |
|  | Not at all or rarely | Sometimes | Often or all the time | Not at all or rarely | Sometimes | Often or all the time | Not at all or rarely | Sometimes | Often or all the time |
| I have trouble remembering what I’ve done yesterday, or conversations I’ve had the previous day. | 0 (0.0) | 6 (28.6) | 15 (71.4) | 2 (6.3) | 8 (25.0) | 22 (68.8) | 12 (48.0) | 8 (32.0) | 5 (20.0) |
| I seem to lack mental energy to get started on activities where I have to pay attention or sustain mental effort. ^a^ | 3 (15.0) | 9 (45.0) | 8 (40.0) | 3 (9.4) | 7 (21.9) | 22 (68.8) | 15 (60.0) | 6 (24.0) | 4 (16.0) |
| Once I get started, I have trouble finishing things. | 7 (33.3) | 5 (23.8) | 9 (42.9) | 8 (25.0) | 10 (31.3) | 14 (43.8) | 16 (64.0) | 4 (16.0) | 5 (20.0) |
| I have trouble keeping my mind on a task for more than a few minutes, like reading or watching TV, even when it's quiet. | 7 (33.3) | 5 (23.8) | 9 (42.9) | 5 (15.6) | 8 (25.0) | 19 (59.4) | 16 (64.0) | 2 (8.0) | 7 (28.0) |
| I get very fatigued during or after activities where I have to pay attention or sustain mental effort. | 2 (9.5) | 6 (28.6) | 13 (61.9) | 7 (21.9) | 5 (15.6) | 20 (62.5) | 14 (56.0) | 5 (20.0) | 6 (24.0) |
| I have trouble adapting to changing task demands throughout the day. | 10 (47.6) | 9 (42.9) | 2 (9.5) | 10 (31.3) | 12 (37.5) | 10 (31.3) | 17 (68.0) | 7 (28.0) | 1 (4.0) |
| I have trouble remembering to do things I said I would, like passing on a message or making an appointment. | 4 (19.0) | 2 (9.5) | 15 (71.4) | 2 (6.3) | 10 (31.3) | 20 (62.5) | 12 (48.0) | 8 (32.0) | 5 (20.0) |
| I have trouble keeping organized throughout the day. | 5 (23.8) | 7 (33.3) | 9 (42.9) | 6 (18.8) | 12 (37.5) | 14 (43.8) | 14 (56.0) | 6 (24.0) | 5 (20.0) |
| I have trouble doing more than one thing at a time. | 5 (23.8) | 4 (19.0) | 12 (57.1) | 1 (3.1) | 12 (37.5) | 19 (59.4) | 14 (56.0) | 6 (24.0) | 5 (20.0) |
| I get overwhelmed by things I have to do. | 8 (38.1) | 5 (23.8) | 8 (38.1) | 7 (21.9) | 11 (34.4) | 14 (43.8) | 17 (68.0) | 4 (16.0) | 4 (16.0) |
| I have trouble following multi-step instructions or keeping track when someone is giving me a lot of details. | 5 (23.8) | 7 (33.3) | 9 (42.9) | 4 (12.5) | 9 (28.1) | 19 (59.4) | 15 (60.0) | 5 (20.0) | 5 (20.0) |
| People have to correct or remind me to get things done. | 4 (19.0) | 9 (42.9) | 8 (38.1) | 5 (15.6) | 15 (46.9) | 12 (37.5) | 18 (72.0) | 4 (16.0) | 3 (12.0) |
| I have trouble getting back on task when I'm interrupted. | 6 (28.6) | 8 (38.1) | 7 (33.3) | 5 (15.6) | 13 (40.6) | 14 (43.8) | 18 (72.0) | 5 (20.0) | 2 (8.0) |
| I feel foggy, like my brain is swimming in molasses. | 5 (23.8) | 10 (47.6) | 6 (28.6) | 11 (34.4) | 9 (28.1) | 12 (37.5) | 18 (72.0) | 5 (20.0) | 2 (8.0) |
| I have trouble focusing on a task in a distracting environment, like background noise or other people talking. | 3 (14.3) | 6 (28.6) | 12 (57.1) | 3 (9.4) | 7 (21.9) | 22 (68.8) | 10 (40.0) | 10 (40.0) | 5 (20.0) |
| I forget how to do routine activities like driving a car. (Bias check) | 17 (81.0) | 3 (14.3) | 1 (5.0) | 29 (90.6) | 3 (9.4) | 0 (0.0) | 25 (100.0) | 0 (0.0) | 0 (0.0) |
| I have trouble finding a word that is on the tip of my tongue. | 1 (4.8) | 7 (33.3) | 13 (61.9) | 4 (12.5) | 11 (34.4) | 17 (53.1) | 13 (52.0) | 8 (32.0) | 4 (16.0) |
| I have trouble getting started, I procrastinate. | 3 (14.3) | 8 (38.1) | 10 (47.6) | 6 (18.8) | 10 (31.3) | 16 (50.0) | 14 (56.0) | 8 (32.0) | 3 (12.0) |
| I can't do things as quickly as I used to, or I make mistakes. | 4 (19.0) | 4 (19.0) | 13 (61.9) | 4 (12.5) | 11 (34.4) | 17 (53.1) | 11 (44.0) | 10 (40.0) | 4 (16.0) |
| I have trouble remembering what I just said, repeating myself in conversations. | 6 (28.6) | 10 (47.6) | 5 (23.8) | 8 (25.0) | 12 (37.5) | 12 (37.5) | 16 (64.0) | 6 (24.0) | 3 (12.0) |
| I am slow to respond when asked a question or when participating in conversations. | 8 (38.1) | 9 (42.9) | 4 (19.0) | 13 (40.6) | 14 (43.8) | 5 (15.6) | 19 (76.0) | 5 (20.0) | 1 (4.0) |
| I have trouble remembering where my everyday items are, like my phone or keys. | 7 (33.3) | 4 (19.0) | 10 (47.6) | 8 (25.0) | 14 (43.8) | 10 (31.3) | 16 (64.0) | 5 (20.0) | 4 (16.0) |
| I have trouble remembering what I’ve just read or what someone just told me. | 3 (14.3) | 9 (42.9) | 9 (42.9) | 3 (9.4) | 14 (43.8) | 15 (46.9) | 13 (52.0) | 7 (28.0) | 5 (20.0) |

*Note*. Data are numbers with percentages in parentheses. ^a^ One active-duty participant did not respond to this question.

## Supplementary Appendix

*Initial questionnaire items with sources*

Source of items:

- Functional Cognition Screen from Compensation Techniques Inventory (Sohlberg & Turkstra, 2011)
- APT-II (Sohlberg, Johnson, Paule, Raskin, & Mateer, 1994)
- Everyday Memory Questionnaire (Royle & Lincoln, 2008)
- Attention Rating and Monitoring Scale (Cicerone, 2002).

Items initially rated as “possibly include":

1. Am slow to respond when asked a question or when participating in conversations (Sohlberg, Johnson, Paule, Raskin, & Mateer, 1994).
2. Needing to repeat things or slow down in order to avoid mistakes (Cicerone, 2002).
3. Seem to lack mental energy to do activities (Sohlberg, 1994).
4. Easily overwhelmed if task has several components (Sohlberg, Johnson, Paule, Raskin, & Mateer, 1994).
5. Becoming irritable or easily overwhelmed due to difficulties paying attention, for example, if you have more than one thing to do or if you are in a noisy environment (Cicerone, 2002).
6. Becoming very fatigued during or after activities where you have to pay attention or sustain mental effort (Cicerone, 2002).
7. Miss details or make mistakes because level of concentration decreased (Sohlberg, Johnson, Paule, Raskin, & Mateer, 1994).
8. Have trouble organizing days and tasks that need to be completed
9. Procrastinating, or having difficulty getting started doing things on your own (Cicerone, 2002).
10. Difficulty maintaining a "train of thought" and keeping things in order, for example, when doing simple calculations in your head or keeping track of several pieces of information.
11. Starting to read something (a book or an article in a newspaper, or a magazine) without realizing you have already read it before (Royle & Lincoln, 2008).
12. Forgetting important details of what you did or what happened to you the day before (Royle & Lincoln, 2008).
13. Forgetting when it was that something happened; for example, whether it was yesterday or last week (Royle & Lincoln, 2008).
14. Difficulty recognizing mistakes until somebody else notices them or points them out (Cicerone, 2002).

Items initially rated as “possibly exclude”:

1. Can’t keep mind on activity or thought because mind feels “spacy” or “blank (Sohlberg, Johnson, Paule, Raskin, & Mateer, 1994).”
2. Difficulty returning to a task after being interrupted (Cicerone, 2002).
3. Being easily distracted by things going on around you (Cicerone, 2002).
4. Can’t keep mind on activity or thought because mind keeps wandering (Sohlberg, Johnson, Paule, Raskin, & Mateer, 1994).
5. Difficulty concentrating in busy or noisy situations, for example, in a shopping center or when more than one person speaking at the same time (Cicerone, 2002).
6. Difficulty concentrating or keeping your mind on an activity for more than a few minutes, for example, when reading or watching television (Cicerone, 2002).
7. Easily get off track if other people milling about nearby (Sohlberg, Johnson, Paule, Raskin, & Mateer, 1994).
8. Easily distracted by surrounding noise (Sohlberg, Johnson, Paule, Raskin, & Mateer, 1994).
9. Trouble paying attention to conversation, if more than one other person (Sohlberg, Johnson, Paule, Raskin, & Mateer, 1994).
10. Easily lose place if task or thinking interrupted (Sohlberg, Johnson, Paule, Raskin, & Mateer, 1994).
11. Difficult to pay attention to more than one thing at a time (Sohlberg, Johnson, Paule, Raskin, & Mateer, 1994).
12. Experiencing (or being told about) periods of time where you are not aware of things going on around you, for example, "staring into space" for several minutes while somebody is talking to you (Cicerone, 2002).
13. Can only concentrate for very short periods of time (Sohlberg, Johnson, Paule, Raskin, & Mateer, 1994).
14. Difficulty doing more than one thing at a time (Sohlberg, Johnson, Paule, Raskin, & Mateer, 1994).
15. Cannot stay focused and return to task when interrupted (Sohlberg & Turkstra, 2011).
16. Lose track of time (Sohlberg & Turkstra, 2011).
17. Start but don’t finish tasks (Sohlberg & Turkstra, 2011).
18. When reading a newspaper or magazine, being unable to follow the thread of a story; losing track of what it is about (Royle & Lincoln, 2008).
19. Having to check whether you have done something that you should have done (Royle & Lincoln, 2008).
20. When talking to someone, forgetting what you have just said. Maybe saying ‘what was I talking about (Royle & Lincoln, 2008)?’
21. Starting to do something and forgetting what you meant to do (Cicerone, 2002).
22. Getting the details of what someone was told you mixed up and confused (Royle & Lincoln, 2008).
23. Repeating to someone what you have just told them or asking someone the same question twice (Royle & Lincoln, 2008).
24. Forget what I did yesterday (Sohlberg & Turkstra, 2011).
25. Lose keys (Sohlberg & Turkstra, 2011).
26. Forgetting to tell somebody something important, perhaps forgetting to pass on a message or remind someone of something (Royle & Lincoln, 2008).
27. Don’t know what appointments are coming up next week (Sohlberg & Turkstra, 2011).
28. Forget to complete tasks at home or work (Sohlberg & Turkstra, 2011).
29. Double schedule (Sohlberg & Turkstra, 2011).
30. Miss appointments (Sohlberg & Turkstra, 2011).
31. Forgetting that you were told something yesterday or a few days ago, and maybe having to be reminded about it (Royle & Lincoln, 2008).
32. Forgetting things immediately or just after being told, for example, phone conversations (Cicerone, 2002).
33. Finding that a word is ‘on the tip of your tongue’. You know what it is but cannot quite find it (Royle & Lincoln, 2008).
34. Completely forgetting to do things you said you would do, and things you planned to do (Royle & Lincoln, 2008).
35. Forgetting where things are normally kept or looking for them in the wrong places (Royle & Lincoln, 2008).

**References**

Cicerone, K.D. (2002) Remediation of ‘working attention’ after mild traumatic brain injury. *Brain Injury*, *16*, 185-195. doi:10.1080/02699050110103959

Royle, J., & Lincoln, N.B. (2008). The everyday memory questionnaire-revised: Development of a 13-item scale. *Disability and Rehabilitation, 30*, 114-121. doi: 10.1080/09638280701223876

Sohlberg, M. M., Johnson, L., Paule, L., Raskin, S. A., & Mateer, C. A. (1994). *Attention Process Training II: A program to address attentional deﬁcits for persons with mild cognitive dysfunction* [rehabilitation materials]. Puyallup, WA: Association for Neuropsychological Research & Development.

Sohlberg, M. M., & Turkstra, L. S. (2011). *Optimizing cognitive rehabilitation: Effective instructional methods.* Location: New York, NY: Guilford Press
